# Dr. Brown-Séquard’s Lectures

**Published:** 1852-06

**Authors:** 


Dr. Brown-Sequard, of Paris, has commenced a course of lectures on Experimental Physiology, in this city, which promises to be highly instructive and interesting. His lectures are illustrated by vivisections, and are intended to establish facts and theories in relation to electro-physiology, and the physiology of the nervous system and of the muscular tissue.

The name of Dr. Brown-Sequard is well known to all who have watched the progress of physiology during the last few years, as an able and enthusiastic cultivator of this department of medical science.

---

Our foreign and domestic retrospect, together with several bibliographical notices, and other matters prepared for this number, are unavoidably postponed to make room for the reports of the American Medical Association, and of the Pennsylvania State Medical Society. They will appear in the July number.

